# Psychiatric diagnosis: the indispensability of ambivalence

**DOI:** 10.1136/medethics-2013-101763

**Published:** 2014-02-10

**Authors:** Felicity Callard

**Keywords:** psychiatric diagnosis, DSM–5, sociology, personal narratives

## Abstract

The author analyses how debate over the fifth edition of the *Diagnostic and Statistical Manual of Mental Disorders* has tended to privilege certain conceptions of psychiatric diagnosis over others, as well as to polarise positions regarding psychiatric diagnosis. The article aims to muddy the black and white tenor of many discussions regarding psychiatric diagnosis by moving away from the preoccupation with diagnosis as classification and refocusing attention on diagnosis as a temporally and spatially complex, as well as highly mediated process. The article draws on historical, sociological and first-person perspectives regarding psychiatric diagnosis in order to emphasise the conceptual—and potentially ethical—benefits of ambivalence vis-à-vis the achievements and problems of psychiatric diagnosis.

## Introduction

‘To some extent every diagnosis brings asylum and stigma.’^1^

In this article, I use the temporal vantage point of the debates surrounding the fifth edition of the *Diagnostic and Statistical Manual of Mental Disorders* (*DSM–5*) to trouble how they have addressed psychiatric diagnosis and privileged certain ethical positions and formulations over others. The article aims to muddy the black and white tenor of many DSM–5 discussions regarding psychiatric diagnosis, to refocus attention on diagnosis as a complex, highly mediated process (in contrast with the preoccupation with diagnosis as classification), and to raise to visibility the potential ethical benefits of remaining ambivalent as regards the achievements and problems of psychiatric diagnosis. I write as a medical humanities scholar who researches 20th and 21st century psychiatry, and also as someone who has received psychiatric diagnoses—and lived, ambivalently, with, through, and perhaps beyond them—in the USA and the UK.

## Exhausting divisions

In the months prior to publication of DSM–5, there was an explosion of print, broadcast and online media discussions. In making my way through these often angry discursive thickets, I reflected on how they tended to prioritise particular kinds of ethical quandaries and positions over others. (For example, the problematics of creeping medicalisation and potential overdiagnosis were very prominent,^2^ ^3^ in comparison with the relative absence of substantive discussion regarding, say, what effects DSM–5 might have in the Global South.) In the process, it became clear that many discussions used a palette of black and white—a palette with exiguous amounts of grey. (Headlines such as ‘DSM–5: a manual run amok’,^4^ ‘The DSM–5 is not crazy’^5^ and ‘Medicine's big new battleground: does mental illness really exist?’^6^ give a taste of the affective intensity and polarisation in the media.) What came to fascinate as well as disturb me was the Manichaean character of these discussions—the battles that attempted to carve good from bad, true from misguided, what should be defended from what should be destroyed. The rhetorical certainty has been striking: many of those critiquing the DSM–5 have had no qualms with making bold judgements such as ‘the medical model is bust’^7^; many of those defending DSM–5 seemed equally convinced of the forward march of science and its incontrovertible benefits for patients. (For example, David Kupfer, Chair of the DSM–5 Taskforce argued: ‘Science will advance and we will learn more about the intersection of brain, genes, environment and behaviour. DSM must reflect that knowledge. Our patients will be better off for it’.)^8^

Much of that Manichaeism is a function of how media are structured to foment disagreements. But that is not the whole story. It is helpful to attend to how publication of the DSM–5 has reanimated earlier, 20th century disputes about the effects of psychiatric diagnosis, the role of psychiatry in society and the so-called ‘(bio)medical model’. Sometimes, it felt as though the debates over DSM–5 had plunged us back into the 1960s and 1970s, the era of intense disagreements between representatives of the psychiatric orthodoxy and those positioned as ‘antipsychiatrists’. There is a complex historicity of disputation within psychiatry: in this case, the DSM–5 has come to function as a highly visible flashpoint in a heterogeneous mental health landscape, through which earlier disputes and epistemological fights can be resurrected. Currently, the temptation seems to have been to caricature and isolate positions that are perceived to be distinct from one's own. This runs counter to what Michael Staub, in his careful reassessment of antipsychiatry in the 1960s and 1970s, has identified as the ‘*mutual entanglements* of psychiatry and antipsychiatry’ (italics added), entanglements that continue up to the present. For example, many powerful social theorisations of madness, as well as critiques of psychiatry, emerged from *within* psychiatry as well as from outside of it; various arguments about and analyses of the role of social processes in the generation of mental disorder criss-crossed psychiatric and social scientific communities, rather than being generated and used solely in one domain or the other; and many so-called ‘anti-psychiatry’ strands in the 1960s and 1970s advocated complex and ambivalent positions regarding the so-called psychiatric establishment, mental illness and the role of diagnosis.^9^ An acknowledgement of these mutual entanglements and of the importance of ambivalence seems in short supply in current DSM–5 debates, even as they revivify older vocabularies and debates from the 1960s and 1970s.

Here are two examples, one taken from the heart of American psychiatry, the other from a piece coauthored by a prominent British clinical psychologist.^i^ The first is by the President of the American Psychiatric Association, Jeffrey Lieberman, and functioned as a defence of DSM–5 and a response to its many critics. Lieberman stated with astonishment that there are ‘groups who are actually proud to identify themselves as “antipsychiatry”’:

These are real people who don't want to improve mental healthcare, unlike the … psychiatrists, psychologists, social workers and patient advocates who have labored for years to revise the DSM, rigorously and responsibly. Instead, they are against the diagnosis and treatment of mental illnesses … and “against” the very idea of psychiatry ... They are, to my mind, misguided and misleading ideologues and self-promoters who are spreading scientific anarchy.^10^

Lieberman, while noting the inadequacies of psychiatric treatment in the era of psychosurgery and insulin shock therapy, could not conceive how there might be any defensible ‘antipsychiatric’ challenge to today's psychiatry of ‘psychopharmacology, neuroimaging, molecular genetics and biology’. Indeed, his argument implicitly framed anyone not labouring to revise the DSM ‘rigorously and responsibly’ as implacably opposed to the improvement of mental healthcare. While there are some organisations that are opposed to ‘the very idea of psychiatry’, it is invidious and incorrect to regard all opposition to the DSM–5 as equivalent to opposition to the improvement of mental healthcare tout court.

The second is by Daniel Freeman (clinical psychologist) and Jason Freeman (writer and editor):

Implicit here is a debate about the nature of mental illness. The DSM uses a medical model of psychiatric illness. It thinks in terms of separate, discrete disorders, just like physical medicine. ….

[T]he *psychological* model of mental illness … [a]rgues that there's no binary opposition between disorder and ‘normality’. Psychological disorders are simply the extreme manifestation of traits that we all possess to varying degrees. …

[T]he *sociological* model … argue[s] that psychological disorders aren't illnesses at all. They're a label used to stigmatize and control behaviour society deems objectionable — such as homosexuality, which featured in the *DSM* until 1980.

Our view is that psychological problems aren't illusory. They are real expressions of distress, for which most people—understandably—want help.^11^

Here we see a binary division being made between those who assert that mental illness is ‘illusory’ (‘the sociological model’, on the Freemans’ account) and those who argue that mental illness is ‘real’ (the psychiatric and the psychological models). The sociological model is sketched as one that regards the ascription of ‘disorder’ as merely a ‘label’ that facilitates stigmatisation and social control. Lieberman's and the Freemans’ accounts are bathed in the waters of older modes of argument and disciplinary expertise, though these are waters distilled from what were originally more complex arguments. Lieberman and the Freemans want, in different ways, to draw a firm line between robust clinical models of mental illness/mental distress and what lies beyond them—whether the so-called antipsychiatrists who are supposedly spreading scientific anarchy, or the sociologists who are supposedly arguing that psychological disorders are ‘labels’ aiding the dubious project of social control.

The attempt to install this dividing line belies many of the characteristics of mental health landscape: (1) there has for a number of decades been significant interpenetration of psychiatric, psychological and sociological styles and modes of thought^12^ ^13^; (2) ‘psychiatry’ is not a singular, discrete entity; rather, as Pickersgill has argued, ‘it is a heterogeneous assemblage of interacting material and symbolic elements’, such that it is unwise to assume that there can be any stable and straightforward critique of its practices and processes *as a whole*^14^; (3) the mental health landscape is traversed by actors, groups and social movements who ally themselves with—and distance themselves from—others in complex configurations that can in no way be adequately captured under the black and white monikers of propsychiatry or antipsychiatry. The emergence of the ‘global mental health movement’, the changing contours of the disability movement, the rise of collaborative research involving clinicians and service user researchers, and the rich tapestry of (ex-) service user movements all require more nuanced accounts of the relations, partnerships and critiques that criss-cross psychiatry and mental health.^15^

However, many discussions regarding DSM–5 and psychiatric diagnosis appear (as indicated by Pickersgill15a) to bracket or occlude these complex histories and current realities. Such a discursive landscape too rapidly renders invisible less certain and more ambivalent positions vis-à-vis psychiatric diagnosis. If such complexity is forgotten or disavowed, it is difficult for ethical analyses to do justice to the range of positions and voices within the broad mental health landscape. In addition, the rich historical archive of philosophical and political debates and disputes over psychiatric diagnosis might well hold resources for current clinical and social scientific research on psychiatric diagnosis: these resources might not be recognised if attention is paid only to more straightforward, one-dimensional accounts of diagnosis.

## Ambivalence and mediation

This article argues, then, for the potential ethical importance of muddier, non-doctrinaire accounts of psychiatric diagnosis. I mean muddier, here, as regards what diagnoses (can) do and (can) not do; as regards who and what drive practices of psychiatric diagnosis; and as regards ambivalence in relation to the uses, achievements and problems of psychiatric diagnosis. My argument is subtended by the research I have conducted relating to psychiatric diagnosis,^16^ ^17^ as well as my own experiences of psychiatric diagnoses. I am convinced that in order to engage in full and robust ethical examination of the DSM–5 (and of other psychiatric nosologies), we need to contend with two important realities that have largely been ignored in the DSM–5 debates. First, many (most?) people relate to psychiatric diagnosis—perhaps particularly so when they have a diagnosis themselves—in ways that are contextually variable, ambivalent and labile. Second, the effects of and responses to psychiatric diagnosis are profoundly *uneven*: they depend on what the actual diagnosis is; who receives it; at what point in her life; whether it is her first or her sixth psychiatric diagnosis; what the particularities of the healthcare systems of the region/country in which she lives are; and what the other axes of identification or ascription are that influence how she is seen by others and/or sees herself.^18^ My emphasis on ‘what the actual diagnosis is’ is intended to draw attention to how unhelpful it is to lump all psychiatric and neurodevelopmental diagnoses into one umbrella category in relation to which normative judgements vis-à-vis the worth of diagnosis are made. It is likely, for example, that the ascription of bipolar 1 (recurrent psychotic mania), schizophrenia and borderline personality disorder diagnoses will carry different consequences, and be greeted in different ways by those receiving them, from the ascription of cyclothymia, depression—or, indeed, autism.

In order adequately to attend to these realities, we need to consider diagnosis as classification (the problematic that has characterised, unsurprisingly, most of the DSM–5 debates) and attend to diagnosis as a process.^19^ ^20^ A focus on process allows us to attend to how diagnostic practices are temporally and spatially complex, and have effects at different scales. Psychiatric diagnosis is not best thought of taking place in a single moment in a single place; it is often not something that ‘matters’ for a diagnosed person in the same way in all the spaces in which she lives her life. Certain psychiatric diagnoses can usher in profound and traumatic events carrying potentially irrevocable consequences (eg, in many countries in Central and Eastern Europe, a diagnosis is used as a ‘status’ ascription (see Szmukler20a) in order to deprive people of their legal capacity, which can lead to long-term institutionalisation).^21^ Certain diagnoses might well serve as the catalyst for poorer treatment in multiple domains (though this might well be the result of compound effects of multiple forms of discrimination and disempowerment, rather than the effect of the psychiatric diagnosis per se.)^22^ While there are many occasions in which dispute about or negotiation around diagnosis (whether by clinicians or by the patient and/or family member) is very common, there are, equally, many other occasions where such negotiation or dispute hardly features. But it is far from clear whether *all* psychiatric diagnoses *always* function as the ‘master status’^23^ that trump other elements of a person's identity. The consequences of psychiatric diagnosis are *heterogeneous*, as are the manifold reactions to them from people who receive them. Psychiatric diagnoses are engaged and lived with in multiple, ambivalent and often contradictory ways.

Exploring diagnosis as process also allows us to discern how deeply and unavoidably mediated processes of psychiatric diagnosis tend to be. My use of the adjective mediated, here, is intended to refer to the general ways in which processes of communication intervene in and fundamentally shape the creation and circulation of meaning, and, more specifically, to what the media theorist Roger Silverstone described as the ‘fundamentally, but unevenly, dialectical process in which institutionalised media of communication (the press, broadcast radio and television, and … the worldwide web), are involved in the general circulation of symbols in social life’.^24^ ^25^ The DSM–5 discussions have tended—implicitly if not explicitly—to portray diagnosis as that which is imposed (by psychiatry, which has the power) on the patient (who does not) (eg, ‘A label of psychiatric illness would … be imposed on people who would fare much better without one’).^26^ But a growing body of sociological and historical research indicates the intricate ways in which (proto)patients and doctors negotiate over which diagnosis (if any) is given; in which people fight to be given particular diagnoses; and, in which people identify with, and take on, particular diagnoses prior to, or outside of, clinical contact.^14^ ^27^ Psychiatric diagnosis might still to a significant extent be enabled by direct, face-to-face encounters with the patient, rather than, as is the case with numerous other diagnoses, by apparatuses and assays. But it is far from being unmediated: a person commonly receives, interprets and forms a relationship with a psychiatric diagnosis via multiple, fragmented routes, many of which do not involve direct encounters with a clinician. These routes often entail complex engagements with various kind of paperwork, with forms of media (including newspapers, internet fora, social media, newspapers, etc), and through discussions with a wide variety of people embedded in different frameworks through which they understand ‘diagnosis’. We need to approach diagnosis not only through a vision of straightforward clinical encounters in which a clinician ‘gives’ a psychiatric diagnosis (and is interpreted as either good or bad for having done so) and a patient, as passive vessel, is thereby entirely marked by that diagnosis (whether for good or for ill).

To substantiate the arguments above, I present brief vignettes from my own experiences of psychiatric diagnosis.

Nota bene: This example (n=1) should be read in the context of all that has been said above. It is only one of many possible experiences of responses to psychiatric diagnosis: I am in no way representative. In particular, the cultural capital that accrues to me through my education, class position and ethnicity, as well as the particular diagnoses and treatments I received, undoubtedly mean my experiences of psychiatric diagnosis are likely to have been more consensual and negotiatory than those of many (particularly many of those receiving psychotic diagnoses and/or involuntary treatment).

## XXX.X

One day, not very long after the publication of DSM–IV,^28^ in the US state of Maryland, I left a psychiatrist's office with two sheets of paper. One was a psychopharmacological prescription; the other a bill to send to my insurance company. Included on the bill was an opaque code. I knew it was a DSM–IV code—I was in the midst of doctoral course work on 20th-century psychiatry—but had no idea what it denoted. I was fascinated by the period after the first three letters. What did the three numbers–full stop–number mean? How, moreover, did they correspond to the narratives I had recounted, the behaviours I had demonstrated and described, and the intense affective states that I had manifested in front of the psychiatrist?

The psychiatrist had not said a word about the diagnosis. At that time, the internet was not yet omnipresent, and so I did what many of us did in those long-lost, pre internet days when one wanted to turn something opaque into something comprehensible: I went into the bowels of a library, pulled out the university's copy of the DSM–IV, and looked up ‘my’ diagnosis. I tried to fit the description of behaviour, affective state, bodily specifics and internal mentation to my own experiences of the days, and months that stretched behind me. *(In other words, what I found helped to frame my recollections of the previous months as well as the experiences I was having in the present; the diagnosis also functioned as an incitement to undertake a series of hermeneutical inquiries.)*

I also filled the pharmacological prescription and read all the accompanying paperwork. I envisaged the side effects that I might shortly experience, which undoubtedly made me more vigilant towards certain parts of my body. That vigilance played a part in identifying—if not in actually helping to materialise—some of the side effects that did, duly arrive. Direct-to- Consumer Advertising on television had not started, and so I did not have those representations of the mentally ill person with ‘my’ diagnosis before and after pharmacological treatment to help model the experience of psychiatric diagnosis. But I talked to fellow graduate students and other friends who had also received psychiatric diagnoses, made rather awkward internet forays (it was the mid-1990s and most of us were still amateurs online) and looked at email discussion lists. I participated, alongside others, in attempts to discern whether similar diagnoses made us similar kinds of people; we wondered whether a single DSM–IV psychiatric diagnosis was capacious enough to allow for what appeared to us to be phenomenologically diverse experiences. *(In other words, a range of processes—the imbibing of pharmacological prescriptions, the conversing over diagnoses online and offline, the comparing of side effects, the telling and retelling of illness histories—helped solidify particular ontologies and phenomenologies of mental illness.)*

When in a subsequent appointment, I tentatively asked the psychiatrist about the code and hence the diagnosis, she simply said, “Don't take it too seriously, I don't.” We never returned to the question of diagnosis again.

## Paperwork

Several years later, when I had moved back to the UK, my general practitioner (GP) referred me into secondary psychiatric care. I never knew what diagnosis I had received from him, though he did say that because I was not responding to the drugs I had been prescribed, the diagnosis might not be correct. *(In other words, here I was told that it might well be the pharmacological interventions, rather than the physician himself, that would be the key actor in achieving the correct diagnosis.)*

While in secondary psychiatric care, I was copied in to various letters being sent about me—and my mental illness—to different people for different purposes. Here is a small selection:

**From the GP to the Head of Department**: ‘I have signed ––– off work for X months owing to [**DIAGNOSIS A**].’

**From the psychiatrist to the GP**: ‘She does indeed continue to experience quite severe, incapacitating symptoms of ZZZ and YYY, with pronounced secondary PPP. …. Overall, my impression is of a severe [**DIAGNOSIS B**], with secondary features of QQQ, which currently do not fulfil diagnostic criteria for [**DIAGNOSIS C**].’

**From the psychiatrist to the consultant psychotherapist**: ‘I am writing to request your opinion in regard of this interesting woman, who suffers with severe, and at times disabling [**DIAGNOSIS D**]’.

**Exchanges between occupational health and department of human resources**: ‘––– has been on long term sick with [**DIAGNOSIS A**]’. ‘She is receiving treatment for her condition and her return to work will depend upon her response to this treatment.’ ‘––– should be regarded as disabled as defined by the Disability Discrimination Act 1995.’ ‘While she will be prone to recurrent illness in the future it is quite likely she will have long periods of remission’.^ii^

**From human resources to head of department**: ‘––– is still signed off work with [**DIAGNOSIS A**].’

What is of interest here? First, the diagnostic terms are used for several distinct purposes: to underpin a medical insurance claim; to justify to an employer why an employee was not at work; to ensure that an employer fulfilled particular duties in relation to an employee; to capture a case history that would later be appended to a larger set of clinical records; to speak across clinical boundaries (here, between psychiatry and psychotherapy) to ascertain if psychotherapy might be appropriate alongside pharmacological treatment; and to inform an employer how an employee was likely to perform in the future. Second, not once has a clinician given a diagnosis directly to me. I have received—if that is the correct verb—several diagnoses through different kinds of paperwork; on none was I the addressee. Diagnostic terms were commonly interwoven—often in complex ways—with the terms ‘condition’, ‘illness’, ‘disorder’ (as well as the language of symptoms). That paperwork traced a disconcerting, fragmentary and highly mediated journey—one that included two psychiatric taxonomic systems, primary and secondary care, and insurance, employment and human resources procedures. It incited in me significant interpretative activity, much of which I conducted outside of clinical spaces. The extent to which the clinicians were committed to the diagnostic terms they used—as opposed to employing them for pragmatic reasons—was far from clear. (I remember, for example, clinical occasions that, retrospectively, I interpreted as the psychiatrist implying that he would mobilise a diagnosis for purposes whose ends he believed we had agreed upon.) These diagnoses were central elements of a set of procedures that recounted my past, presented others with aspects of what I was like in the present, and prescribed me with a likely future. These diagnoses undoubtedly marked me, and marked my life. But there are two, crucial additional points: First, I in no way simply internalised what was documented in this paperwork: my affective responses to these various diagnostic terms have included puzzlement, relief, recognition, anger, curiosity, reassurance and alienation (often several at once), and they have varied significantly over a number of years. Second, I am emphatically *not* arguing that all would have been straightforward if the clinicians were to have spoken to me face to face about these diagnoses (nor, in fact, that I would have preferred them to have done so). Rather, I want to insist on two points, which I argue are generalisable across multiple psychiatric diagnoses and in relation to the majority of people who receive diagnoses. (1) Experiences of psychiatric diagnosis are indispensably mediated: even if one's clinician speaks extensively face to face about the diagnosis, there is *always* a much wider set of discourses and traces that frame the meanings of the diagnosis; and (2) there are many layers of interpretation that lie between the bald diagnostic category and the person who is positioned in relation to it (ie, one should not a priori assume that one knows how the person so designated by that term might take it up or reject it).

## Conclusion

The DSM–5 discussions have tended to be polarised, and have tended to present streamlined accounts of what a psychiatric diagnosis is and whether the DSM–5 taxonomy is normatively good or bad. Within this polarised landscape, diagnosis has commonly been conceptualised as an event that is driven by psychiatry, and that is discrete, punctual, and that ‘captures’ (for good or ill) the person so designated. In contrast, this article calls for greater attention to the messiness and diversity of reactions to and positions regarding DSM–5, and of the configurations through which psychiatry and mental health are made and remade. I have argued, in particular, that we need to keep in clear view greyer, more ambivalent accounts of psychiatric diagnosis. By this, I mean accounts that attend to how diagnosis comprises processes and practices that are highly mediated across time and across space, and that incur temporally and spatially heterogeneous and ambivalent responses from those who receive them.

Those espousing certainty in relation to psychiatric diagnosis (whichever end of the spectrum they lie) have tended to grab much of the discursive space through which ethical issues surrounding diagnosis might be deliberated, and have created an environment—whether deliberately or not—in which a limited number of concerns are commonly regarded *as the central ethical issues* concerning psychiatric diagnosis. This is, in itself, a matter of ethical concern. For example, current emphasis (in light of the DSM–5 debates) on diagnosis as classification rather than as process has arguably deflected energy away from focused consideration of if, when, how and by whom negotiation over particular diagnoses does—and indeed *should*—take place. The underacknowledgement of diagnosis as a highly mediated set of practices has arguably resulted in a lack of critical reflection on how the communication of psychiatric diagnoses takes place (and indeed *should* take place). If, for example, clinicians are no longer imagined as the primary conduit through which a diagnosis is given—but rather as one node in a complex network of influences, transmitters, and cultural atmospheres that shape understandings of psychiatric diagnosis—how might this affect the role of the clinician in communicating and discussing psychiatric diagnoses? Or if every act of psychiatric diagnosis is understood to be embedded in complex—and very particular—social, legal, clinical, cultural, familial and psychological configurations, how might this affect the act of making normative judgements about whether diagnosis is ‘good’ or ‘bad’?

There are, in addition, potential ethical values and benefits that might be more easily opened up through ambivalence than through (premature) certainty.^29^ These include the possibility of delaying rather than rushing to judgement; of attending to voices in these debates that are currently seldom or never heard; of disaggregating clinicians’ use of psychiatric diagnoses from any necessary agreement with, or endorsement of those diagnoses; of more capacious and balanced interrogations of the achievements, limits and uses of psychiatric diagnosis; and, finally, of attempting to doing conceptual and epistemic justice^30^ to the multifaceted ways in which those in receipt of psychiatric diagnosis live with, through, against and beyond them.

## Footnotes

i There are a large number of examples that I could have chosen. My selection was governed by my wish to indicate how influential voices of those with clinical expertise—as well as the voices of journalists—have contributed to polarising and/or caricaturing positions within the DSM–5 discussions.

ii In these exchanges, the language slips between ‘diagnosis’, ‘condition’ and ‘illness’.
